# Biomimetic Nanoparticles Loaded With α‐Cyperone Alleviating LPS‐Induced Inflammation in KGN Cells by Activating Nrf2/HO‐1 and Suppressing ROS

**DOI:** 10.1002/jbt.70495

**Published:** 2025-09-18

**Authors:** Jialing Li, Fengzhi Li, Xue Chen, Jie Ma, Hua Guo

**Affiliations:** ^1^ Reproductive Medicine Center General Hosptial of Ningxia Medical University Yinchuan China; ^2^ The Fifth People's Hospital of Ningxia Hui Autonomous Region Yinchuan China; ^3^ Ningxia Medical University Yinchuan China; ^4^ Department of Gynecology General Hospital of Ningxia Medical University Yinchuan China

**Keywords:** antioxidant, diminished ovarian reserve, granulosa cells, inflammation, nanocomplexes, α‐cyperone

## Abstract

Diminished ovarian reserve (DOR) is a leading cause of female infertility, and currently, no effective therapeutic options are available. α‐Cyperone (AC) possesses various pharmacological properties, including anti‐inflammatory and antioxidant effects. However, its clinical application is hindered by poor water solubility, a short half‐life, and nonspecific toxicity. In this study, we utilized nanotechnology to develop a novel dual‐targeted nanocomplex, termed PLGA@AC@FSHL‐M (PAMF) nanoparticles (NPs), comprising poly(lactic‐co‐glycolic acid) (PLGA) encapsulating AC and camouflaged with a macrophage membrane modified by the FSHL81‐95 peptide. This design enabled efficient delivery of AC while simultaneously targeting granulosa cells (GCs). Our findings demonstrated that PAMF NPs significantly reduced the production of tumor necrosis factor‐α (TNF‐α), interleukin‐6 (IL‐6), and interleukin‐1β (IL‐1β) in lipopolysaccharide (LPS)‐induced KGN cells. Furthermore, AC‐loaded PAMF NPs enhanced nuclear translocation of nuclear factor erythroid 2–related factor 2 (Nrf2) and upregulated heme oxygenase‐1 (HO‐1), while inhibiting NF‐κβ activation. These results suggest that biomimetic AC‐loaded nanoparticles effectively suppress apoptosis and promote proliferation under inflammatory conditions in KGN cells, offering a promising therapeutic strategy for DOR.

## Graphical abstract

In this study, we developed a novel dual‐targeted nanocomplex, which not only overcame the limitations of α‐Cyperone but also achieved simultaneous targeting of granulosa cells. Biomimetic nanoparticles loaded with α‐Cyperone effectively suppressed apoptosis and promoted proliferation of LPS‐induced inflammation in KGN cells, thereby offering a promising therapeutic strategy for DOR.

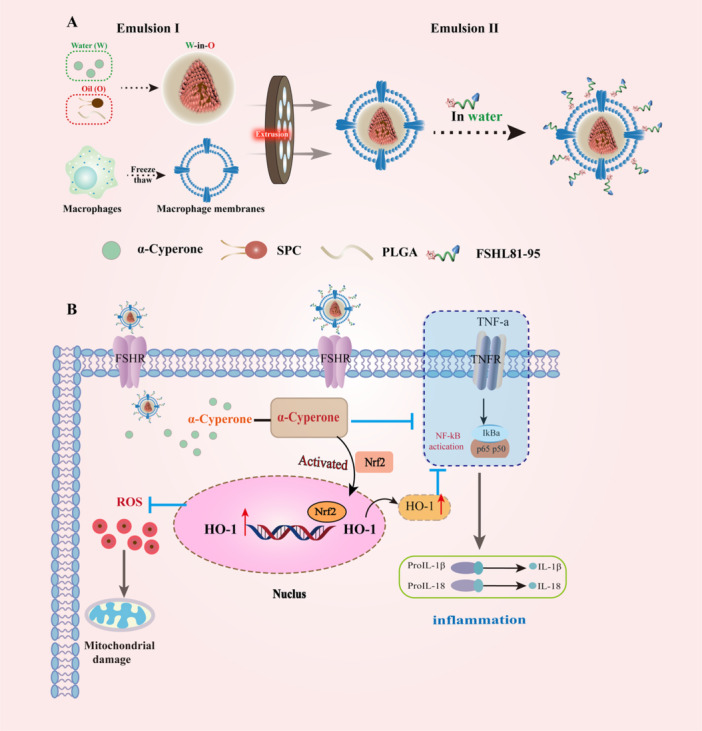

## 1 Introduction

Diminished ovarian reserve (DOR) is a prevalent condition associated with female reproductive aging, significantly impacting both fertility and quality of life in affected women [1]. This condition compromises reproductive potential and is increasingly recognized to involve multifactorial etiologies, including genetic, iatrogenic, immunological, and environmental factors [2, 3]. Among these, oxidative stress (OS), inflammation, and mitochondrial dysfunction have emerged as critical contributors to the underlying pathophysiological mechanisms of aging‐related reproductive decline [4, 5].

Granulosa cells (GCs) are essential for oocyte nourishment and directly influence oocyte quality. Elevated OS in GCs is regarded as a key factor leading to their functional impairment [6, 7]. Mitochondria, maternally inherited double‐membraned organelles, generate ATP via oxidative phosphorylation (OXPHOS) and concurrently produce reactive oxygen species (ROS). Under physiological conditions, ROS are integral to nuclear maturation and act as signaling molecules in folliculogenesis, oocyte maturation, and ovulation [8, 9]. However, excessive ROS accumulation can induce mitochondrial dysfunction in GCs, decrease ATP production, and disrupt the meiotic spindle apparatus required for chromosome segregation [10]. Consequently, oxidative/antioxidant imbalance is considered a major cause of GC damage, ultimately contributing to the onset of DOR [11].

Chronic inflammation is another contributing factor that adversely affects ovarian cellular function, leading to impaired ovarian reserve and fertility decline [5, 12]. Persistent inflammatory states can disrupt GC function, resulting in hormonal imbalances and defective follicular development. This, in turn, may impair reproductive health. Inflammatory responses are closely linked to OS, forming a vicious cycle in which inflammation exacerbates oxidative damage, thereby accelerating cellular injury and disease progression [13].

Cyperi Rhizoma (Xiangfu), a traditional Chinese medicinal herb, has a long history of clinical use. Modern pharmacological research has identified a‐Cyperone (AC) as its major bioactive constituent, exhibiting diverse biological effects, including antioxidant, anti‐inflammatory, antitumor, antibacterial, and hypoglycemic activities [14]. For instance, AC has been shown to protect cardiomyocytes from inflammation and OS induced by oxygen‐glucose deprivation [15]. However, the potential therapeutic effect of AC in LPS‐induced inflammation in KGN cells remains unexplored. Moreover, its oily and volatile nature significantly limits its clinical application. Therefore, the development of a targeted drug delivery system for AC holds great potential for improving ovarian function.

**Scheme 1:**
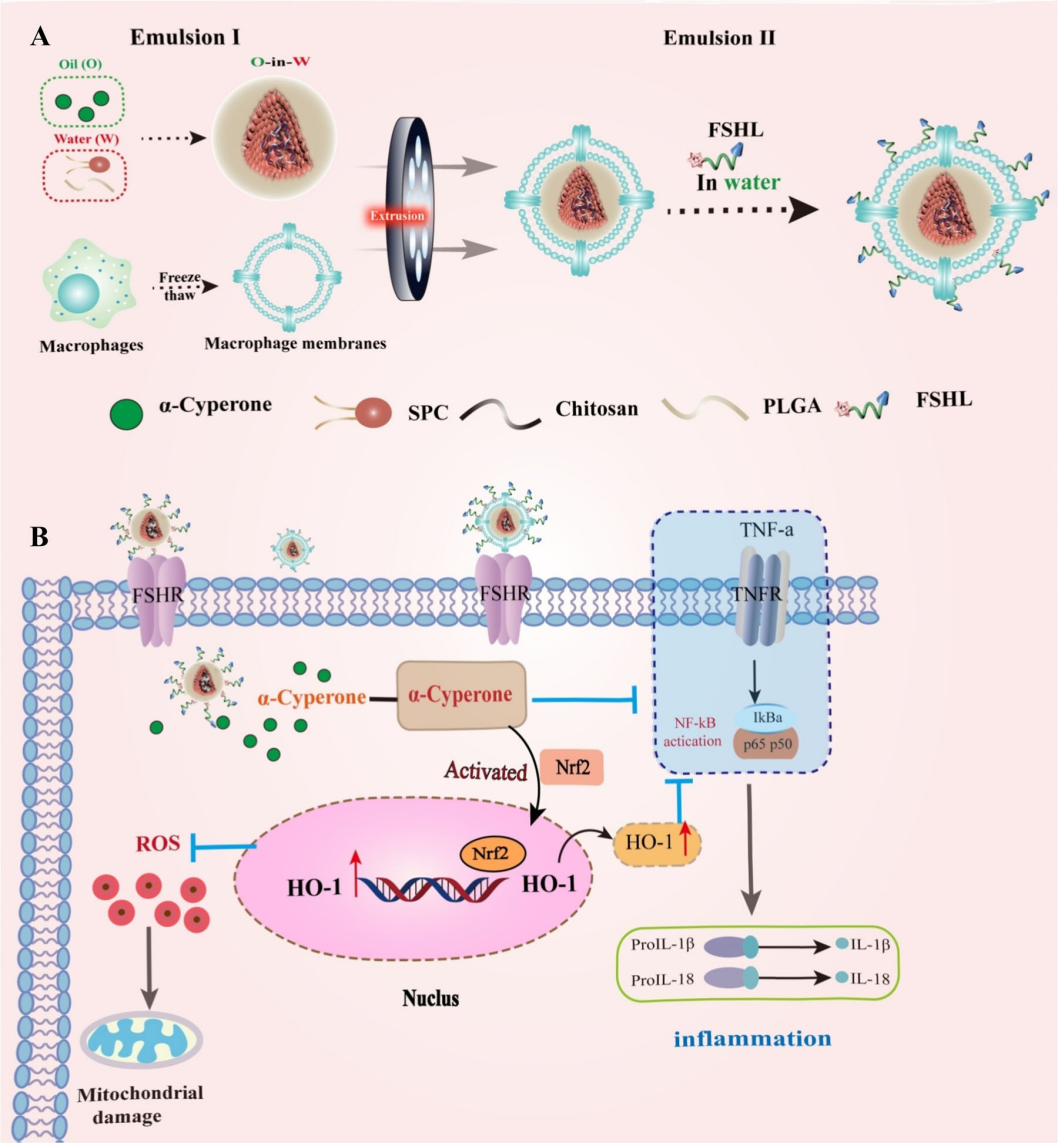
Schematic illustration of the administration of PAMF for DOR. (A) The synthetic process of FMPA NPs. (B) The acidic‐responsive released AC could effect to anti‐inflammatory, antioxidant and apoptosis.

In the present study, we designed an innovative AC‐loaded nanosystem (illustrated in Scheme 1A) aimed at dual‐targeting GCs to enhance therapeutic outcomes for DOR (Scheme 1B). AC was encapsulated into PLGA to form PLGA@AC (PA) NPs. These were subsequently coated with a macrophage‐derived membrane (M), generating PLGA@AC@M (PAM) NPs to prolong circulation time. To achieve selective targeting of GCs, the FSHL81‐95 (QCHCGKCDSDSTDCT) peptide, which specifically binds to follicle‐stimulating hormone receptors (FSHR) highly expressed in GCs, was conjugated to the membrane surface, yielding PLGA@AC@FSHL‐M (PAMF) NPs.

This biomimetic nanosystem (PAMF NPs) modulated immune responses and attenuated inflammatory hyperplasia through combined anti‐inflammatory, antioxidant, and antiapoptotic mechanisms. Consequently, this platform conferred robust protection to GCs and holds considerable promise as a potent therapeutic approach for treating DOR.

## 2 Materials and Methods

### 2.1 Materials and Reagents

AC was purchased from Master of Bioactive Molecules (Shanghai, China). Monoclonal antibodies against IL‐6, IL‐1β, and TNF‐α, along with the TB Green® Premix Ex Taq™ II kit (Code No. RR820A), were obtained from Qinke Biochemical Co. Ltd. (Jiangsu, China). Antibodies against β‐actin and Nrf2 were procured from Annuolun Biotechnology Co. Ltd. (Beijing, China). A membrane protein extraction kit and DAPI were purchased from Beyotime (Shanghai, China). Hoechst 33342 and propidium iodide (PI) kits were acquired from Yeasen Biotech Co. Ltd. (Shanghai, China). The CCK‐8, ROS, and BCA protein assay kits were obtained from TargetMol (MA, USA), and all other reagents and chemicals were of analytical grade. MitoTracker® Red CMXRos (#9082) was purchased from Cell Signaling Technology (USA). PLGA (50:50, MW: 38,000–54,000), 1‐(3‐Dimethylaminopropyl)‐3‐ethylcar‐bodiimide hydrochloride (EDC), N‐Hydroxysuccinimide (NHS), and imethyl sulfoxide (DMSO) were acquired from Sigma‐Aldrich (USA). Polyvinyl alcohol (PVA, MW: 9000–10,000) and dichloromethane (DCM) were obtained from Shanghai Macklin Biochemical Co. Ltd. Pharmaceutical‐grade chitosan oligosaccharide (molecular weight: 1.2 kDa, 95% deacetylation) was purchased from Zhejiang Golden Shell Biochemical Co. Ltd. (Zhejiang, China).

### 2.2 Cells Line

KGN cells were obtained from Procell Life Technology Co. Ltd. (Wuhan, China). The cells were cultured in Dulbecco's Modified Eagle Medium (DMEM) supplemented with 10% fetal bovine serum (FBS) and 1% penicillin‐streptomycin (PS) (Invitrogen, Carlsbad, CA, USA). All cultures were maintained at 37°C in a humidified incubator with 5% CO₂.

### 2.3 Preparation of Macrophage Membrane (M)

Macrophage membranes were isolated from RAW264.7 mouse cells using a membrane protein extraction kit (Beyotime, China). Briefly, membrane protein extraction reagent A was added to the RAW264.7 cells and incubated at 4°C for 20 min. The cell suspension was then disrupted using an ultrasonic cell pulverizer (40 kHz) for 10 min. After undergoing repeated freeze‐thaw cycles at −80°C and 37°C, the lysate was centrifuged at 700 rpm for 10 min to remove cellular debris, followed by a second centrifugation at 14,000 rpm for 30 min to isolate the macrophage membranes.

### 2.4 Synthesis of FSHL81‐95 Peptide

The FSHL81‐95 peptide [sequence (N → C): (PEG₈)‐(PEG₈)‐(PEG₈)‐QCHCGKC DSDSTDCT; CAS: 04010067493] was synthesized by Qiangyao Biotechnology Co. Ltd. (Shanghai, China).

### 2.5 Preparation and Characterization of SPMF NPs

PA NPs were synthesized via a nanoprecipitation technique. Briefly, PLGA (10 mg) and AC (1 mg) were dissolved in DMSO and sonicated for 5 min to form the oil phase. This solution was added dropwise into 5% PVA under magnetic stirring, followed by dialysis for 12 h using a dialysis bag with a molecular weight cut‐off (MWCO) of 2500 Da.

To prepare macrophage membrane‐coated nanoparticles (PAM NPs), the macrophage membrane and PA NPs were mixed and incubated at 37°C for 2 h. The resulting PAM NPs were then combined with FSHL81‐95 peptide at a 2:1 (w/w) ratio in PBS and stirred at 37°C for 12 h to obtain the final PAMF NPs.

The size distribution and zeta potential of the nanoparticles were measured using a Nano ZS Zetasizer (Malvern, UK). For morphological analysis, nanoparticles were placed onto a copper grid, stained with 2.0% phosphotungstic acid, air‐dried, and visualized under a transmission electron microscope (TEM; Hitachi, Tokyo, Japan). The encapsulation efficiency (EE) of AC was calculated using the following equation:

EE(%)=(AmountofACloadedinPLGA/InitialPLGAamount)×100%.

### 2.6 Encapsulation Efficiency and In Vitro Release of AC

To assess the AC encapsulation efficiency, a standard curve of free AC‐FAM (absorbance vs. concentration) was constructed using a FL‐2500 spectrophotometer (Hitachi, Japan) with excitation/emission wavelengths of 257/518 nm. This curve was used to determine the AC‐FAM concentration in PAMF NP samples.

For release studies, PAMF NP solution was placed in a dialysis bag (MWCO: 3.5 kDa) and immersed in PBS at pH 7.4 and pH 5.4, maintained at 37°C with agitation at 400 rpm. At predetermined time points (0, 6, 12, 18, 24, 30, and 36 h), samples were centrifuged at 10,000 rpm for 10 min, and the supernatant was analyzed using the FL‐2500 spectrophotometer (Ex/Em = 257/518 nm). The cumulative release rate of AC was calculated using the following formula:

CumulativeACrelease(%)=Mt/Mo×100%

where Mt is the amount of AC released at each time point, and Mo is the initial amount of AC encapsulated. Each experiment was performed in triplicate.

### 2.7 Quantitative Real‐Time Polymerase Chain Reaction (qRT‐PCR)

Quantitative real‐time PCR was conducted using the TB Green® Premix Ex Taq™ II Kit according to the manufacturer's instructions. A standard two‐step amplification protocol was employed. The thermal cycling program began with an initial denaturation at 95°C for 30 s, followed by 40 cycles at 95°C for 5 s and 60°C for 30 s (Table 1).

**TABLE 1:** PCR primers for target genes.

Gene	Primer sequence (5′ to 3′)	
IL‐1β	Forward primer	TTCGAGGCACAAGGCACAACAG
	Reverse primer	TAGTGGTGGTCGGAGATTCGTAGC
IL‐6	Forward primer	TGAGAGTAGTGAGGAACAAGCCAGAG
	Reverse primer	TGACCAGAAGAAGGAATGCCCATTAAC
TNF‐a	Forward primer	AGAGGGAGAGAAGCAACTACAGACC
	Reverse primer	AGGAAGGAGAAGAGGCTGAGGAAC

### 2.8 Western Blot

Cells were lysed using RIPA buffer, and the resulting protein samples were subjected to Western blot analysis. Proteins were separated by electrophoresis using 10% and 12% SDS‐PAGE gels and transferred onto PVDF membranes (Massachusetts, USA). The membranes were then blocked with 5% milk in TBST and incubated overnight at 4°C with specific primary antibodies: IL‐6 (1:1000), IL‐1β (1:1000), TNF‐α (1:1000), Nrf2 (1:2000), and β‐actin (1:10000). Following this, the membranes were incubated with the appropriate secondary antibodies (goat anti‐rabbit or goat anti‐mouse) at a dilution of 1:2000 for 2 h. Detection reagents were applied to the PVDF membranes (0.22 μm and 0.45 μm), and the protein bands were visualized using the Bio‐Rad ChemiDoc XRS Chemiluminescence System.

### 2.9 Enzyme Linked Immunosorbent Assay (ELISA)

Following treatment, the culture supernatant was collected and centrifuged. The concentrations of TNF‐α, IL‐1β, and IL‐6 were measured using human‐specific ELISA kits according to the manufacturer's instructions. Absorbance was recorded at a wavelength of 450 nm.

### 2.10 Cellular Proliferation Analysis

Cell proliferation was assessed using the MTT assay. KGN cells were seeded in 96‐well plates and incubated at 37°C for 36 h before being treated with PBS, LPS (10 μg/mL), or AC at concentrations of 0, 20, 40, 80, and 120 μM for 24 h. Cell viability was subsequently determined using the Cell Counting Kit‐8 (CCK‐8), and absorbance was measured at 570 nm with a reference wavelength of 630 nm using an iMark Absorbance Spectrophotometer (Bio‐Rad, USA).

### 2.11 Cellular Immunofluorescence

KGN cells were cultured in 24‐well plates at 37°C for 48 h and then treated with PBS, LPS, AC, PA, MPA, or FMPA (AC at 20 μM; LPS at 10 μg/mL) for another 48 h. After fixation with paraformaldehyde and permeabilization with 0.1% Triton X‐100, cells were blocked with goat serum for 2 h. Slides were incubated with rabbit monoclonal anti‐Nrf2 antibody (1:200), followed by incubation with a secondary anti‐rabbit antibody (1:500). Intracellular fluorescence (green signal) was observed using a confocal laser scanning microscope (CLSM).

### 2.12 ROS Assay and Mitochondrial Function Experiment

Intracellular ROS levels were measured using 2,7‐dichlorofluorescein diacetate (DCFH‐DA). KGN cells were seeded at a density of 1 × 10⁵ cells per well in 12‐well plates and treated with PBS, LPS, AC, PA, PAM, or PAMF for 48 h (AC at 20 μM; LPS at 10 μg/mL). After treatment, cells were incubated with serum‐free medium containing 10 μM DCFH‐DA at 37°C for 30 min. Excess DCFH‐DA was removed by washing three times with PBS, and fluorescence was immediately observed under a fluorescence microscope.

For mitochondrial function analysis, KGN cells were seeded in 24‐well plates at 1 × 10⁵ cells per well and incubated for 48 h. Cells were treated with PBS, LPS, AC, PA, MPA, or FMPA (AC at 20 μM; LPS at 10 μg/mL). Subsequently, they were incubated with 200 nM MitoTracker Red CMXRos at 37°C for 20 min. After three PBS washes to remove excess dye, cells were immediately observed using a fluorescence microscope.

### 2.13 Statistical Analysis

All data are presented as the mean ± standard deviation (SD) from independent experiments. Student's unpaired two‐tailed *t*‐test was used for comparisons between two groups, while one‐way ANOVA was employed for multiple group comparisons. Statistical analyses were performed using GraphPad Prism 10 software (GraphPad Software, CA, USA). Statistical significance was denoted as follows: *p < 0.05, **p < 0.01, and ***p < 0.001.

## 3 Results

### 3.1 The Impact of AC on LPS‐Induced Inflammation in KGN Cells

**FIGURE 1:**
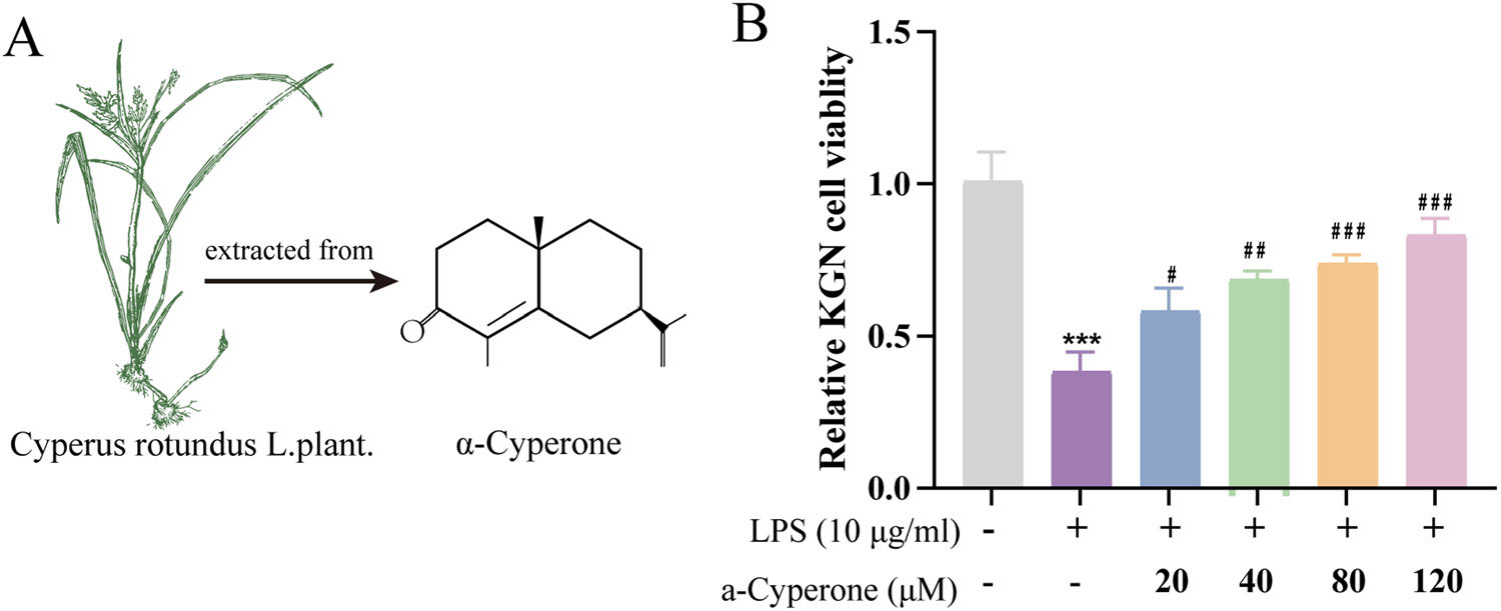
Structure of AC and MTT of KGN cells with different AC NPs groups. (A) Structure of AC and *Cyperus rotundus* L. plant [17, 18]. (B) Relative KGN cell viability levels in different groups. ***p < 0.001 versus the control group; ^#^ *p* < 0.05, ^##^ *p* < 0.01,^###^ *p* < 0.001 versus the LPS group.

AC has demonstrated both antioxidant and anti‐inflammatory properties [16]; however, its effects on KGN cells remain unclear. To assess the potential protective effects of AC against LPS‐induced inflammation in KGN cells, cell viability was evaluated using the MTT assay. Statistical analysis revealed significant differences in cell viability among the various AC concentrations (20, 40, 80, 160 μM) and LPS (10 μg/mL, Figure S1) treatment groups (Figure 1). Cell viability increased significantly with rising concentrations of AC, with 80 μM identified as the optimal concentration.

**FIGURE 2:**
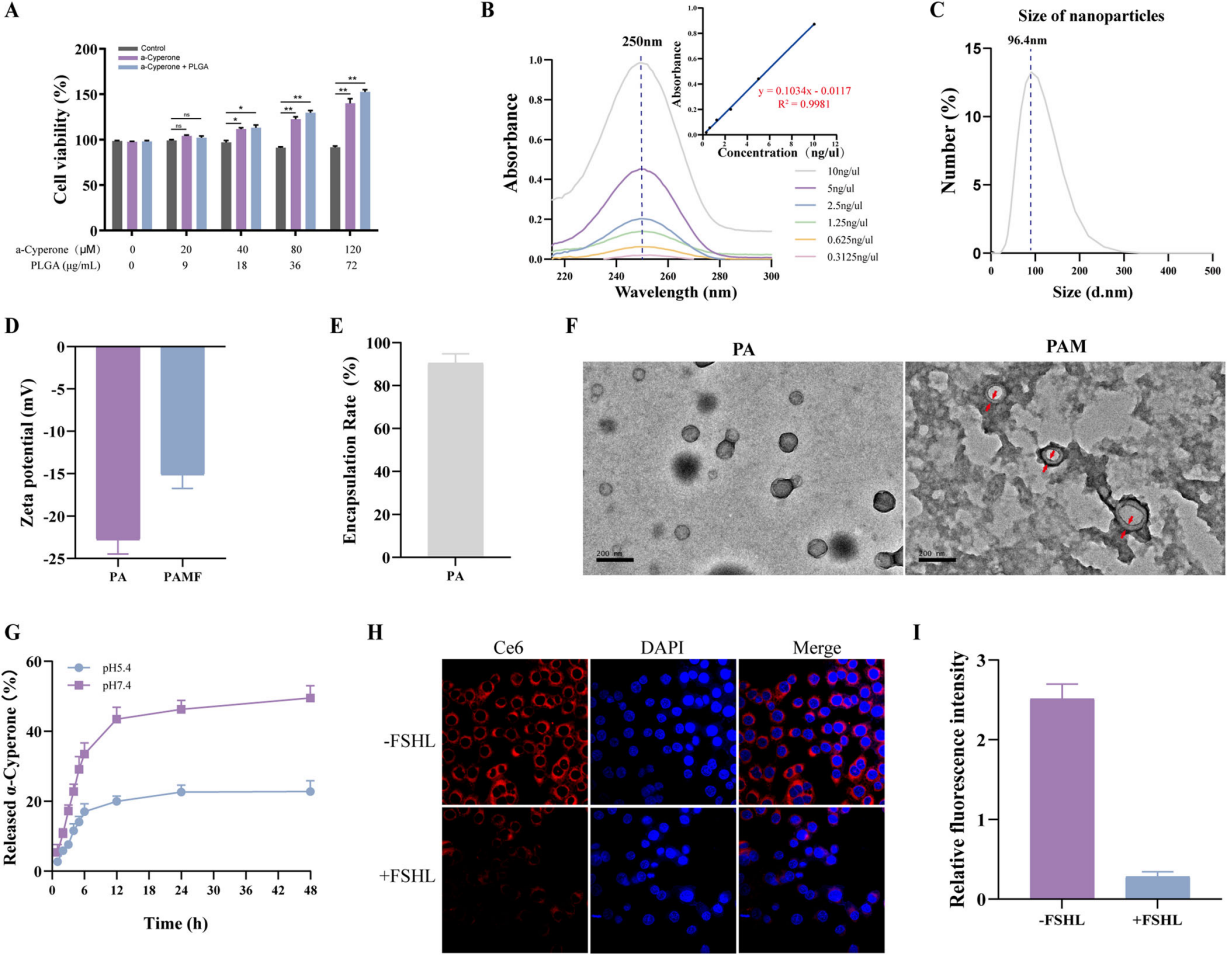
Preparation and characterization of PAMF NPs. (A) Effect of AC and PLGA on cell viability with different concentration ratio. (B) The fluorescence emission spectra and standard curves of free AC. (C, D) Particle size and ζ‐potential of PA NPs. (E) Encapsulation rate of AC in PA NPs. (F) TEM image of AP and PAM NPs. (G) In vitro drug release profile investigation of AC from PAM NPs (pH 7.4 and pH 5.4). (H, I) CLSM images of KGN cells incubation with PAMF NPs with/without FSHL81‐95 peptide fragments pretreatment and relative florescence intensity analysis. *p < 0.05, **p < 0.01.

Considering both drug concentration and efficacy, the optimal combination was determined to be PLGA at 36 μg/mL and AC at 80 μM (Figure 2A). Based on fluorescence absorbance measurements and standard curves of free AC, the characteristic absorption peak at 250 nm was markedly quenched (Figure 2B). Following encapsulation of AC in PLGA, the resulting nanoparticles (PA NPs) exhibited an average diameter of approximately 98 nm and a zeta potential of −22.4 mV (Figure 2C,D), confirming successful AC loading.

The encapsulation efficiency of AC in PA NPs was calculated to be 89.4% (Figure 2E). To enhance nanoparticle circulation time and improve in vivo drug targeting, a membrane camouflage strategy was employed. This approach retained the nanoparticles' drug‐loading capacity and preserved the advantages of their nanoscale size [19]. Accordingly, biomimetic macrophage membrane‐coated, AC‐loaded nanoparticles (PAM NPs) were successfully synthesized.

### 3.2 Synthesis and Identification of AC‐Loaded Biomimetic Nanoparticles

Due to the volatility, poor water solubility, short half‐life, and nontargeted toxicity of cyperone [20], its therapeutic efficacy is limited. To address these limitations, we developed AC‐loaded biomimetic nanoparticles. Initially, the MTT assay was employed to assess the effects of PA nanoparticles (PA NPs) on cell viability. As illustrated in Figure 2A, PA NPs enhanced the proliferation of KGN cells (ranging from 107.2% to 141.6%) within the concentration range of 20 μM to 120 μM. As a result of the proton sponge effect [21], PAM NPs can be engineered for controlled release in response to pH fluctuations within the tissue microenvironment, thereby enabling intracellular drug release. To examine the pH‐mediated regulation of drug release, we investigated the release profile of AC from PAM NPs. As shown in Figure 2G, AC exhibited a time‐ and pH‐dependent release pattern, with 19.2% of AC released over 48 h under pH 7.4, and 45.7% released under pH 5.4. Meanwhile, we also detected the long‐term stability of the nanoparticles (Figure S2) and their metabolic pathways in vivo (Figure S3).

The FSHR is specifically expressed on granulosa cells in the ovary, making it a promising target for ovarian drug delivery [22]. To effectively deliver AC to ovarian granulosa cells, the FSHL81‐95 peptide, which selectively binds to FSHR, was used to modify the surface of PAMF NPs. The internalization efficiency of PAMF NPs was further evaluated in KGN cells pre‐incubated with the FSHL81‐95 polypeptide for 2 h. As shown in Figure 2H–L, pretreatment with FSHL81‐95 significantly impeded the cellular uptake of PAMF NPs, reducing internalization efficiency by approximately eightfold. These results indicate that the presence of the FSHL81‐95 polypeptide facilitates the cellular entry of PAMF NPs by specifically targeting FSHR on the surface of KGN cells.

### 3.3 Effects of PAMP NPs on Proliferation and Antiapoptotic Activity in LPS‐Induced KGN Cells

**FIGURE 3:**
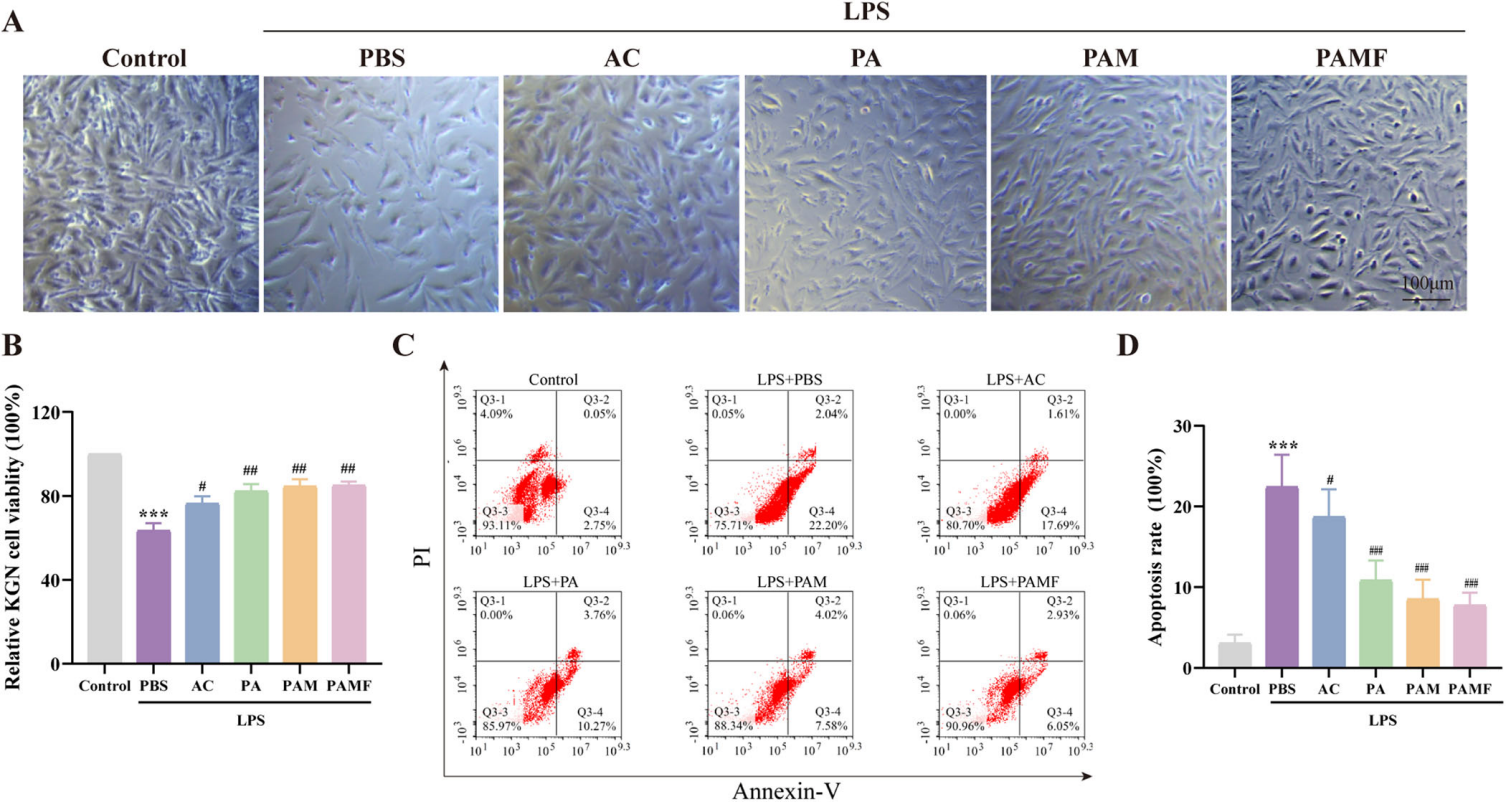
Effect of PAMF NPs on proliferation and antiapoptosis of LPS‐induced KGN cells in vitro. (A, B) Relative ability to proliferation ability in different groups. (C, D) Relative ability to antiapoptotic ability in different groups. ***p < 0.001 versus the control group, ^#^ *p* < 0.05, ^##^ *p* < 0.01, ^###^ *p* < 0.001 versus the LPS + PBS group.

Granulosa cells are essential for maintaining ovarian function, and any dysfunction may compromise reproductive capacity [23]. Therefore, we assessed the effects of PSMF treatment on the proliferation of LPS‐induced KGN cells using the MTT assay. As shown in Figure 3A,B, LPS treatment significantly reduced the proliferation of KGN cells (*p* < 0.001). However, treatment with AC, particularly in the PA, PAM, and PAMF formulations, significantly enhanced cell viability compared to the LPS group (*p* < 0.01).

To further evaluate the antiapoptotic effects of AC‐based treatments on LPS‐induced KGN cells, flow cytometry was performed to determine the apoptosis rate. After 24 h of LPS exposure, the apoptotic rate of KGN cells increased to approximately 24.24% (Figure 3C,D). Treatment with PA, PAM, and PAMF NPs markedly attenuated LPS‐induced apoptosis, suggesting that these formulations protect against LPS‐induced cell death. Notably, PAMF NPs reduced the proportion of Annexin V‐positive/PI‐positive apoptotic cells to approximately 8.98%, a significantly lower rate compared to PA and PAM groups.

### 3.4 Effects of PAMF NPs on Inflammation Suppression in LPS‐Induced KGN Cells

The inflammatory microenvironment can alter granulosa cell growth, differentiation, and hormone secretion, thereby disrupting follicular development and ovulation and adversely affecting reproductive health [24]. To evaluate the anti‐inflammatory potential of PAMF NPs in LPS‐stimulated KGN cells, we measured pro‐inflammatory cytokine levels following different treatments.

**FIGURE 4:**
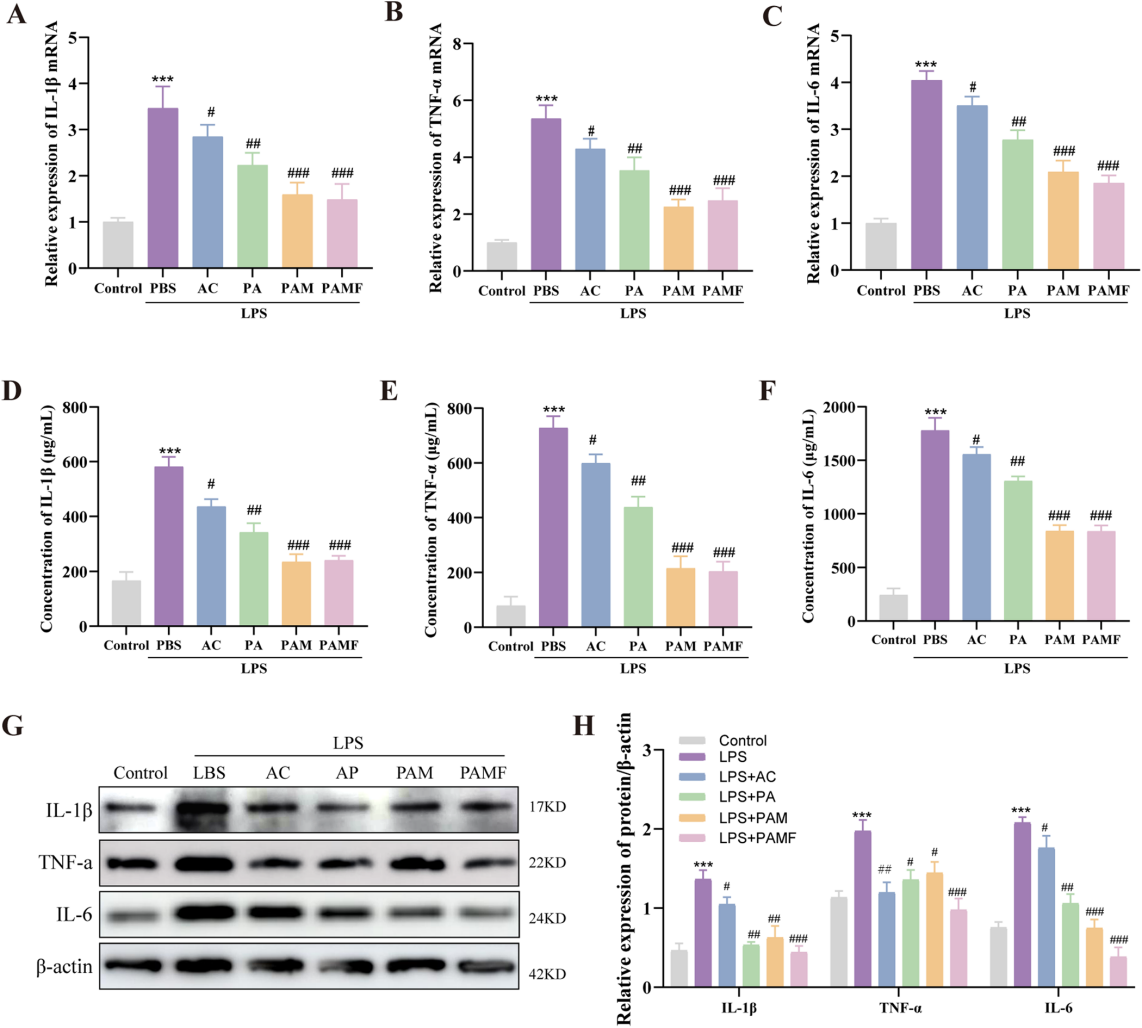
Effects of PAMF NPs on production of pro‐inflammatory cytokines (TNF‐α, IL‐6 and IL‐1β) in LPS‐induced KGN cells. (A–C) The mRNA levels of TNF‐α, IL‐6 and IL‐1β of KGN cells with different treatments and quantitative analysis (real‐time PCR). (D–F) The levels of TNF‐α, IL‐6 and IL‐1β with different treatments and quantitative analysis (the commercial ELISA kits) in culture medium. (G–H) The protein levels of TNF‐α, IL‐6 and IL‐1β with different treatments and quantitative analysis (Western blot) in KGN cells. ***p < 0.001 versus the control group, ^#^ *p* < 0.05, ^##^ *p* < 0.01, ^###^ *p* < 0.001 versus the LPS + PBS group.

After a 24‐h LPS pretreatment followed by 24 h of nanoparticle treatment, the mRNA expression levels of TNF‐α, IL‐6, and IL‐1β were quantified using real‐time PCR, while their protein levels were determined by ELISA. Compared to the control group, the LPS group exhibited significantly elevated mRNA and protein levels of TNF‐α, IL‐6, and IL‐1β, indicating an activated inflammatory response (Figure 4A–F). However, all treatment groups significantly suppressed the expression of these pro‐inflammatory cytokines. Western blot analysis further confirmed that PA, PAM, and PAMF treatments downregulated the protein expression levels of TNF‐α, IL‐6, and IL‐1β in LPS‐treated KGN cells, with PAMF NPs showing the most pronounced inhibitory effect (*p* < 0.001) (Figure 4G–H).

### 3.5 Effects of PAMF NPs on ROS Production in LPS‐Induced KGN Cells

Inflammatory processes can lead to increased intracellular levels of ROS, contributing to oxidative stress and impairing cellular function [25]. ROS, in conjunction with inflammation, modulate cell apoptosis, inflammatory signaling, and other vital cellular processes. Physiological events such as ovulation, elevated oxygen exposure, aging, and mitochondrial dysfunction collectively contribute to ROS generation in ovarian tissues. Excessive ROS accumulation can damage essential biomolecules, thereby disrupting cellular functions and metabolic activities [26].

**FIGURE 5:**
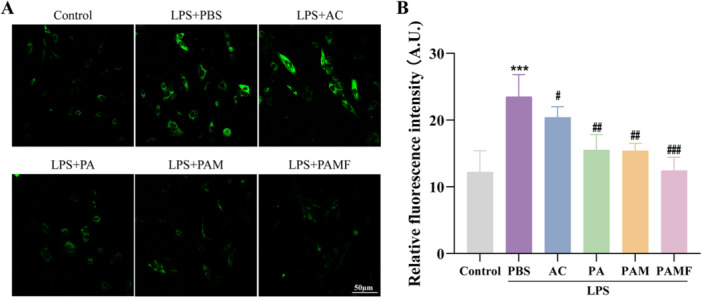
Effects of PAMF NPs on ROS Production in LPS‐induced KGN cells. (A, B) Representation images of ROS levels detected by DCFH staining of KGN cells with different treatments and quantitative analysis. ***p < 0.001 versus the control group, ^#^ *p* < 0.05, ^##^ *p* < 0.01, ^###^ *p* < 0.001 versus the LPS group.

To assess the impact of PAMF NPs on ROS levels in LPS‐induced KGN cells, ROS production was measured using a fluorescence‐based ROS detection kit. KGN cells were pretreated with LPS for 24 h, followed by 24‐h exposure to different treatments. The LPS group exhibited the highest ROS levels (Figure 5A,B, *p* < 0.001). However, treatment with AC—particularly in the PAMF NP group—significantly reduced ROS levels compared to the LPS group (*p* < 0.001).

### 3.6 The Effects of PAMF NPs on Mitochondrial Function in LPS‐Induced KGN Cells

Mitochondria serve as the primary source of ROS in mammalian cells. Excessive ROS accumulation may activate the intrinsic mitochondrial apoptotic pathway, leading to irreversible cellular damage and death [27]. Therefore, we investigated whether PAMF NPs could improve mitochondrial function in KGN cells under inflammatory conditions induced by LPS. Mitochondrial function was assessed using the MitoTracker Deep Red FM assay, a cell‐permeable far‐red fluorescent probe that selectively labels biologically active mitochondria.

**FIGURE 6:**
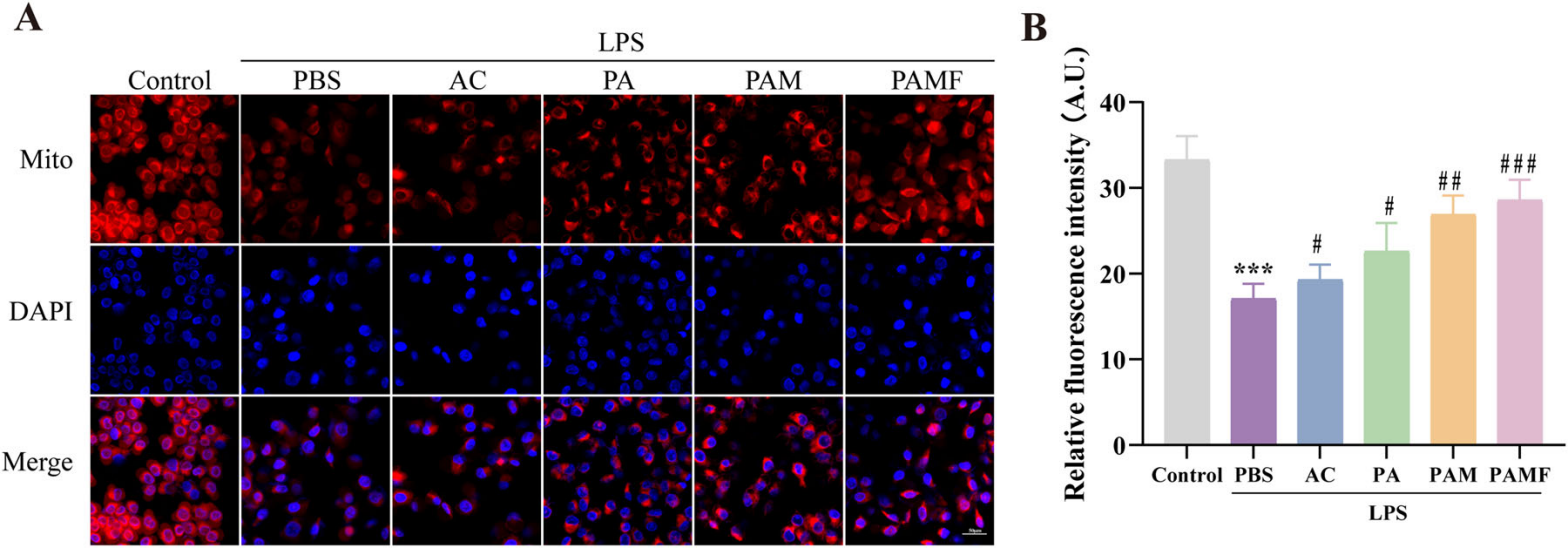
Effects of PAMF NPs on mitochondria function in LPS‐induced KGN cells. (A, B) Relative ability to protect‐Mitochondria ability in vitro of KGN cells with different treatments and quantitative analysis. ***p < 0.001 versus the control group, ^#^ *p* < 0.05, ^##^ *p* < 0.01, ^###^ *p* < 0.001 versus the LPS + PBS group.

The results revealed a significant impairment in mitochondrial function in the LPS group (Figure 6A,B, *p* < 0.001). Following 24 h of treatment with different AC‐loaded formulations, all AC‐treated groups showed significant improvement in mitochondrial function, with the PAMF NPs group exhibiting the most pronounced effect (*p* < 0.001). These findings indicate that AC can enhance mitochondrial function and protect KGN cells from OS‐induced DNA damage.

### 3.7 The Effects of PAMF NPs on Upregulation of HO‐1 Expression and Enhancement of Nrf2 Nuclear Translocation Mediated by the NF‐κB Pathway in LPS‐Induced KGN Cells

**FIGURE 7:**
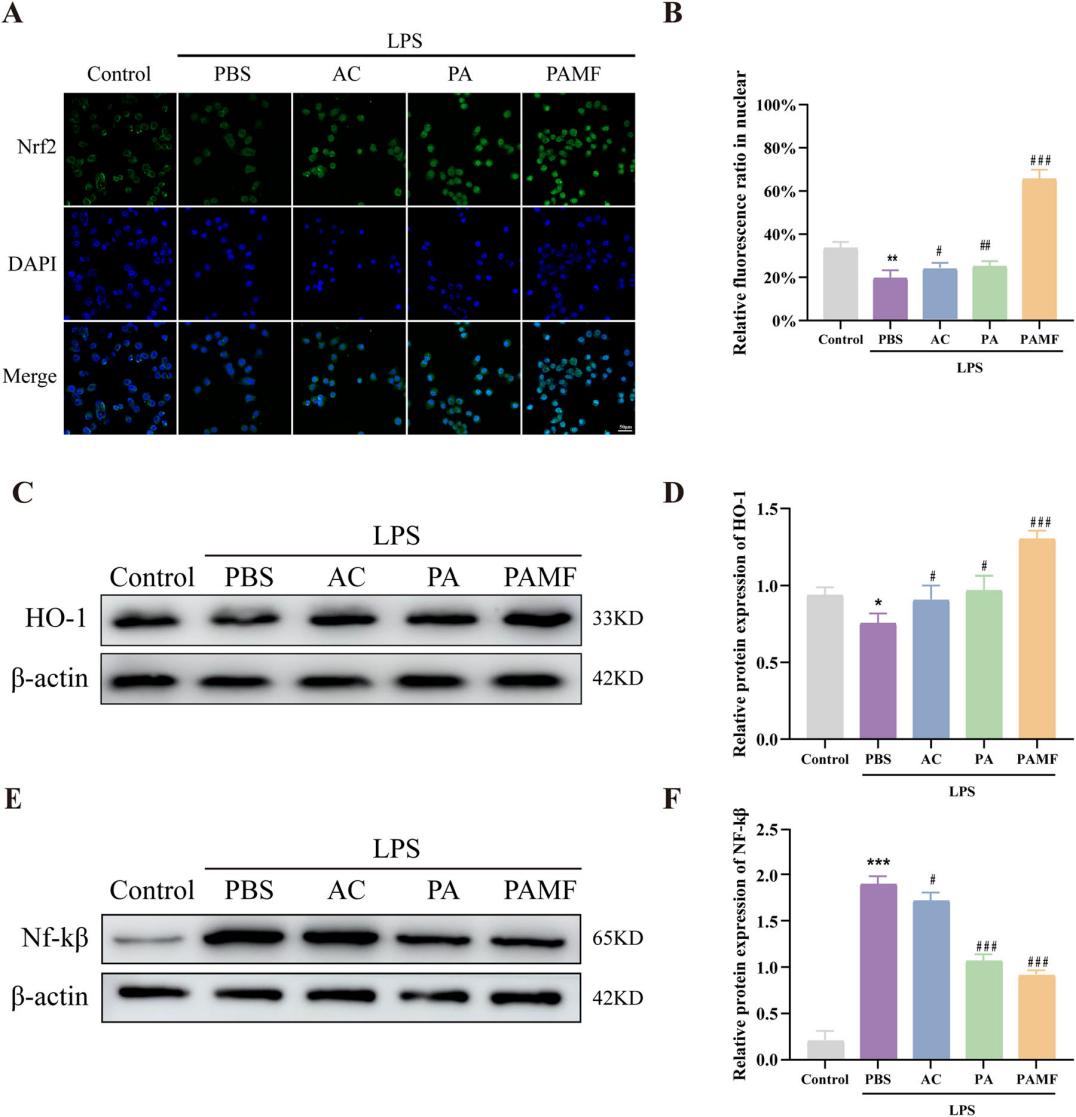
Effects of PAMF NPs upregulates HO‐1 expression and enhances nuclear translocation of Nrf2 mediate by NF‐κβ pathway in LPS‐induced KGN cells. (A, B) Immunofluorescence image of Nrf2 into the nucleus of KGN cells with different treatments and quantitative analysis. (C–F) The protein levels of HO‐1 and NF‐κβ with different treatments and quantitative analysis (Western blot) in KGN cells. *p < 0.05, **p < 0.01, ***p < 0.001 versus the control group, ^##^ *p* < 0.01, ^###^ *p* < 0.001 versus the LPS + PBS group.

AC, a compound derived from traditional Chinese medicine, has been shown to possess antioxidative and anti‐inflammatory properties [28]. Previous studies have demonstrated that AC enhances the antioxidant defense capacity of cells by activating the Nrf2 signaling pathway [29]. To determine whether AC influences Nrf2 nuclear translocation, immunofluorescence staining was conducted. The results indicated that PAMF NPs promoted a progressive increase in Nrf2 nuclear translocation and a corresponding reduction in its cytoplasmic presence in KGN cells exposed to LPS (Figure 7A,B, *p* < 0.001).

NF‐κB plays a key role in mediating inflammatory responses and is rapidly activated by various stimuli, including TNF‐α, IL‐1β, LPS, and ROS [30, 31, 32]. To further investigate the anti‐inflammatory mechanism of PAMF NPs, KGN cells were treated with different AC formulations, and the protein levels of NF‐κB and HO‐1 were analyzed via Western blotting. The results showed that LPS significantly reduced HO‐1 expression (*p* < 0.05) and increased NF‐κB expression (*p* < 0.001). However, treatment with AC formulations significantly reversed these effects, particularly in the PAMF NPs group, which demonstrated the highest HO‐1 expression and lowest NF‐κB expression (Figure 7C–F, *p* < 0.001).

These results suggest that PAMF NPs activate the Nrf2/HO‐1 signaling axis and suppress the NF‐κB pathway, thereby reducing the production of pro‐inflammatory cytokines in LPS‐stimulated KGN cells.

## 4 Discussion

GCs, also known as gamete‐supporting cells, provide the essential nutrients required for oocyte growth and maturation [33]. During follicular development, the proliferation of GCs and their secretion of follicular fluid are critical for the formation of sinusoidal (Graafian) follicles. The amplification and functional differentiation of GCs are fundamental for folliculogenesis and overall ovarian function. However, aberrant GC regulation can impair oocyte competence in women with DOR [34].

Accumulating evidence suggests that altered inflammatory status and OS in GCs significantly contribute to the pathophysiology of DOR [35, 36, 37]. One contributing factor to ovarian aging is ROS accumulation, which is associated with impaired mitochondrial function [38]. Moreover, recent studies indicate that ROS‐induced endoplasmic reticulum (ER) stress plays a decisive role in determining cellular fate, including autophagy, apoptosis, and senescence [39]. Chronic ER stress and accumulation of misfolded proteins are considered key drivers in the manifestation of aging phenotypes [40]. DOR is one of the most prevalent reproductive aging disorders. Whether mitochondrial dysfunction‐induced ROS accumulation in GCs leads to cellular senescence and consequent impairment of ovarian function remains to be fully elucidated.

KGN cells are widely used as a model for studying ovarian disorders. Several antioxidant therapies have been explored for their potential to prevent or treat DOR [41, 42]. Xiangfu (Cyperi Rhizoma), a traditional Chinese medicine, has long been applied in reproductive healthcare [43].

AC, an active component of *Cyperus rotundus*, has shown anti‐inflammatory, antioxidant, and antitumor activities in multiple disease models [16, 29, 44]. Its therapeutic effects continue to be explored. For instance, AC has been reported to alleviate spinal cord injury in rats by activating the Akt/Nrf2 pathway and inhibiting NF‐κB signaling [16, 45]. In psychiatric disorders such as depression, AC exhibits antidepressant potential, possibly through suppression of SIRT3/ROS‐mediated NLRP3 inflammasome activation [46]. However, the role of AC in reproductive medicine, particularly its effects on KGN cells, remains underexplored.

Our findings demonstrate that AC promotes KGN cell proliferation, with the optimal concentration being 40 μM, consistent with prior studies [41, 44]. Due to its volatility, poor aqueous solubility, short half‐life, and off‐target toxicity, the clinical application of cyperone is limited [17]. To overcome these challenges, we developed AC‐loaded biomimetic nanoparticles, offering a more targeted and advanced therapeutic approach. MTT assays confirmed that AC significantly enhanced KGN cell proliferation (111.2%–142.6%), with optimal concentrations of 40 μM for AC and 35 μg/mL for PLGA.

FSHR is specifically expressed on ovarian GCs and has emerged as a potential target for ovarian‐specific delivery strategies [47, 48]. To achieve targeted delivery of AC to ovarian GCs, a FSHL81‐95 peptide—capable of specifically binding to FSHR—was employed to modify the surface of PAM nanoparticles, resulting in the synthesis of PAMF NPs. Our findings demonstrated that PAMF NPs exhibited the most pronounced effect on KGN cell proliferation, attributable to the targeting function of the FSHL81‐95 peptide. These results confirm that PAMF NPs significantly enhanced anti‐inflammatory effects by inhibiting the production and secretion of pro‐inflammatory cytokines (TNF‐α and IL‐6), as well as by attenuating ROS levels, thereby improving mitochondrial function in LPS‐induced KGN cells. These observations are consistent with previously reported antioxidant properties of cyperone [49, 50].

Despite the comprehensive nature of this study, several limitations must be acknowledged. First, our investigation focused primarily on the anti‐inflammatory and antioxidative properties of AC in granulosa cells. Although prior studies have identified the Nrf2/HO‐1 signaling pathway as a classical mechanism associated with AC activity, it is possible that AC may exert protective effects on GCs through alternative signaling pathways implicated in the pathophysiology of DOR, which warrants further exploration. Second, our current work was limited to in vitro cell experiments and did not include in vivo animal studies. Future research will involve animal models to validate the therapeutic potential of AC in improving ovarian function and fertility in the context of DOR. Additional clinical and translational research is essential before these findings can be applied in a clinical setting. Lastly, our study focused solely on the anti‐inflammatory and antioxidant capacities of AC in enhancing GC activity; further investigations are needed to determine whether AC has a direct effect on oocyte function. In conclusion, although additional research is required to fully elucidate the role of AC in the treatment of DOR, our current findings offer novel insights into this therapeutic strategy.

## 5 Conclusion

Recent studies have highlighted the potent anti‐inflammatory, antioxidative, and mitochondrial protective effects of AC in KGN cells, primarily through the activation of the Nrf2 signaling pathway. Moreover, AC significantly suppressed the expression of key inflammatory cytokines, including IL‐1β, IL‐6, and TNF‐α, via a common signaling mechanism. These important findings underscore the therapeutic potential of AC, revealing that its proliferative effect on GCs may be mediated by its regulation of inflammatory mediators. Based on these results, AC may represent a promising therapeutic agent for the treatment of diminished ovarian reserve.

## Author Contributions

Conceptualization: Hua Guo and Jialing Li. Data Curation: Hua Guo and Fengzhi Li. Funding Acquisition: Hua Guo and Jialing Li. Methodology: Jialing Li, Fengzhi Li, Jie Ma, and Xue Chen. Supervision: Hua Guo. Writing – Original Draft: Jialing Li and Fengzhi Li. Writing – Review and Editing: Xue Chen and Jie Ma.

## Ethics Statement

This study was approved by the Ethics Committee of General Hospital of Ningxia Medical University (Ethics review No.KYLL‐2023‐0468).

## Consent

The authors have nothing to report.

## Conflicts of Interest

The authors declare no conflicts of interest.

## Supporting information


**Figure S1:** Relative KGN cell viability levels in different LPS groups.

**Figure S2:** The long‐term stability of PA NPs.

**Figure S3:** In vivo metabolic pathways of PA NPs.

supmat.

## Data Availability

All datasets used and/or analyzed during this study are available from the corresponding author upon reasonable request.

## References

[1] X. Jiao , T. Meng , Y. Zhai , et al., “Ovarian Reserve Markers in Premature Ovarian Insufficiency: Within Different Clinical Stages and Different Etiologies,” Frontiers in Endocrinology 12 (2021): 601752. 33815272 10.3389/fendo.2021.601752 PMC8015703

[2] A. D. Greene , G. Patounakis , and J. H. Segars , “Genetic Associations With Diminished Ovarian Reserve: A Systematic Review of the Literature,” Journal Of Assisted Reproduction And Genetics 31 (2014): 935–946. 24840722 10.1007/s10815-014-0257-5 PMC4130940

[3] N. Li , W. Xu , H. Liu , et al., “Whole Exome Sequencing Reveals Novel Variants Associated With Diminished Ovarian Reserve in Young Women,” Frontiers in Genetics 14 (2023): 1154067. 37065482 10.3389/fgene.2023.1154067 PMC10095150

[4] Z. Zhu , W. Xu , and L. Liu , “Ovarian Aging: Mechanisms and Intervention Strategies,” Medical Review 2 (2022): 590–610. 37724254 10.1515/mr-2022-0031 PMC10471094

[5] X. Zhang , Y. Zhang , X. Chen , P. Qiao , and L. Zhang , “Assessment of the Ovarian Reserve by Serum Anti‐Müllerian Hormone in Rheumatoid Arthritis Patients: A Systematic Review and Meta‐Analysis,” International Archives of Allergy and Immunology 183 (2022): 462–469. 34929705 10.1159/000520133

[6] X. Lin , Y. Dai , X. Tong , et al., “Excessive Oxidative Stress in Cumulus Granulosa Cells Induced Cell Senescence Contributes to Endometriosis‐Associated Infertility,” Redox Biology 30 (2020): 101431. 31972508 10.1016/j.redox.2020.101431 PMC6974790

[7] X. Wang , J. Yang , H. Li , et al., “Corrigendum to “miR‐484 Mediates Oxidative Stress‐Induced Ovarian Dysfunction and Promotes Granulosa Cell Apoptosis via SESN2 Downregulation” [Redox Biol. 62 (2023)1‐16 102684],” Redox Biology 70 (2024): 103084. 38368161 10.1016/j.redox.2024.103084 PMC10907861

[8] K. Shkolnik , A. Tadmor , S. Ben‐Dor , N. Nevo , D. Galiani , and N. Dekel , “Reactive Oxygen Species Are Indispensable in Ovulation,” Proceedings of the National Academy of Sciences 108 (2011): 1462–1467. 10.1073/pnas.1017213108 PMC3029775 21220312

[9] A. Rizzo , M. Roscino , F. Binetti , and R. Sciorsci , “Roles of Reactive Oxygen Species in Female Reproduction,” Reproduction in Domestic Animals 47 (2012): 344–352. 22022825 10.1111/j.1439-0531.2011.01891.x

[10] M. Zhang , M. B. Bener , Z. Jiang , et al., “Mitofusin 2 Plays a Role in Oocyte and Follicle Development, and Is Required to Maintain Ovarian Follicular Reserve During Reproductive Aging.” Aging (Albany NY) (2019). 11, 3919–3938. 31204316 10.18632/aging.102024 PMC6628992

[11] J. Xia , Z. Wang , W. Zou , W. Jin , G. Wu , and J. Yang , “8‐oxoguanine DNA Glycosylase (OGG1) May be a Diagnostic Indicator of Diminished Ovarian Reserve (DOR),” Combinatorial Chemistry & High Throughput Screening 26 (2023): 1058–1065. 35638284 10.2174/1386207325666220527102318

[12] M. Lopez‐Corbeto , S. Martínez‐Mateu , A. Pluma , et al., “The Ovarian Reserve as Measured by the Anti‐Müllerian Hormone Is Not Diminished in Patients With Rheumatoid Arthritis Compared to the Healthy Population,” Clinical and Experimental Rheumatology 39 (2021): 337–343. 32896242

[13] G. Yang , C. J. Wright , M. D. Hinson , et al., “Oxidative Stress and Inflammation Modulate Rev‐erbα Signaling in the Neonatal Lung and Affect Circadian Rhythmicity,” Antioxidants & Redox Signaling 21 (2014): 17–32. 24252172 10.1089/ars.2013.5539 PMC4048579

[14] F. Wang , S. Zhang , J. Zhang , and F. Yuan , “Systematic Review of Ethnomedicine, Phytochemistry, and Pharmacology of Cyperi Rhizoma,” Frontiers in Pharmacology 13 (2022): 965902. 36278199 10.3389/fphar.2022.965902 PMC9585201

[15] F. Yao and Q. Zhu , “Alpha‐Cyperone Protects Cardiomyocytes Against Oxygen‐Glucose Deprivation‐Induced Inflammation and Oxidative Stress by Akt/FOXO3a/NF‐kappaB Pathway,” Disease Markers 2022 (2022): 8205707. 36072899 10.1155/2022/8205707 PMC9444414

[16] M. Deng , P. Xie , J. Liu , et al., “α‐Cyperone Improves Rat Spinal Cord Tissue Damage via Akt/Nrf2 and NF‐κB Pathways,” Journal of Surgical Research 276 (2022): 331–339. 35427911 10.1016/j.jss.2022.02.006

[17] A. M. Pirzada , H. H. Ali , M. Naeem , et al., “Traditional Uses, Phytochemistry, and Pharmacological Activities,” Journal of Ethnopharmacology 174 (2015): 540–560. 26297840 10.1016/j.jep.2015.08.012

[18] A. Kamala , S. K. Middha , and C. S. Karigar , “Plants in Traditional Medicine With Special Reference to *Cyperus rotundus* L.: A Review,” 3 Biotech 8 (2018): 309. 10.1007/s13205-018-1328-6 PMC6037646 30002998

[19] Y. Zhang , K. Cai , C. Li , et al., “Macrophage‐Membrane‐Coated Nanoparticles for Tumor‐Targeted Chemotherapy,” Nano Letters 18 (2018): 1908–1915. 29473753 10.1021/acs.nanolett.7b05263 PMC7470025

[20] P. Liu , E. Shang , Y. Zhu , D. Qian , and J. Duan , “Volatile Component Interaction Effects on Compatibility of Cyperi Rhizoma and Angelicae Sinensis Radix or Chuanxiong Rhizoma by UPLC‐MS/MS and Response Surface Analysis,” Journal of Pharmaceutical and Biomedical Analysis 160 (2018): 135–143. 30086506 10.1016/j.jpba.2018.07.060

[21] P. Neuberg and A. Kichler , “Recent Developments in Nucleic Acid Delivery With Polyethylenimines,” Advances in Genetics 88 (2014): 263–288. 25409609 10.1016/B978-0-12-800148-6.00009-2

[22] A. Perales‐Puchalt , N. Svoronos , M. R. Rutkowski , et al., “Follicle‐Stimulating Hormone Receptor Is Expressed by Most Ovarian Cancer Subtypes and Is a Safe and Effective Immunotherapeutic Target,” Clinical Cancer Research 23 (2017): 441–453. 27435394 10.1158/1078-0432.CCR-16-0492 PMC5241180

[23] H. Li , X. Wang , H. Mu , et al., “Mir‐484 Contributes to Diminished Ovarian Reserve by Regulating Granulosa Cell Function via YAP1‐Mediated Mitochondrial Function and Apoptosis,” International Journal of Biological Sciences 18 (2022): 1008–1021. 35173533 10.7150/ijbs.68028 PMC8771835

[24] A. D. Horlock , T. J. R. Ormsby , M. J. D. Clift , J. E. P. Santos , J. J. Bromfield , and I. M. Sheldon , “Cholesterol Supports Bovine Granulosa Cell Inflammatory Responses to Lipopolysaccharide,” Reproduction 164 (2022): 109–123. 35900358 10.1530/REP-22-0032

[25] M. Mittal , M. R. Siddiqui , K. Tran , S. P. Reddy , and A. B. Malik , “Reactive Oxygen Species in Inflammation and Tissue Injury,” Antioxidants & Redox Signaling 20 (2014): 1126–1167. 23991888 10.1089/ars.2012.5149 PMC3929010

[26] M. Valko , D. Leibfritz , J. Moncol , M. T. D. Cronin , M. Mazur , and J. Telser , “Free Radicals and Antioxidants in Normal Physiological Functions and Human Disease,” International Journal of Biochemistry & Cell Biology 39 (2007): 44–84. 16978905 10.1016/j.biocel.2006.07.001

[27] C. H. Wang , S. B. Wu , Y. T. Wu , and Y. H. Wei , “Oxidative Stress Response Elicited by Mitochondrial Dysfunction: Implication in the Pathophysiology of Aging,” Experimental Biology and Medicine 238 (2013): 450–460. 23856898 10.1177/1535370213493069

[28] J. Lu , W. Li , T. Gao , S. Wang , C. Fu , and S. Wang , “The Association Study of Chemical Compositions and Their Pharmacological Effects of Cyperi Rhizoma (Xiangfu), a Potential Traditional Chinese Medicine for Treating Depression,” Journal of Ethnopharmacology 287 (2022): 114962. 34968659 10.1016/j.jep.2021.114962

[29] B. Huang , D. He , G. Chen , et al., “α‐Cyperone Inhibits LPS‐Induced Inflammation in BV‐2 Cells Through Activation of Akt/Nrf2/HO‐1 and Suppression of the NF‐κB Pathway,” Food & Function 9 (2018): 2735–2743. 29667667 10.1039/c8fo00057c

[30] L. Osborn , S. Kunkel , and G. J. Nabel , “Tumor Necrosis Factor Alpha and Interleukin 1 Stimulate the Human Immunodeficiency Virus Enhancer by Activation of the Nuclear Factor Kappa B,” Proceedings of the National Academy of Sciences 86 (1989): 2336–2340. 10.1073/pnas.86.7.2336 PMC286907 2494664

[31] Y. Kida , M. Kobayashi , T. Suzuki , et al., “Interleukin‐1 Stimulates Cytokines, Prostaglandin E2 and Matrix metalloproteinase‐1 Production via Activation of MAPK/AP‐1 and NF‐κB in Human Gingival Fibroblasts,” Cytokine 29 (2005): 159–168. 15652448 10.1016/j.cyto.2004.10.009

[32] H. Qin , C. A. Wilson , S. J. Lee , X. Zhao , and E. N. Benveniste , “LPS Induces CD40 Gene Expression Through the Activation of NF‐κB and STAT‐1α in Macrophages and Microglia,” Blood 106 (2005): 3114–3122. 16020513 10.1182/blood-2005-02-0759 PMC1895321

[33] J. J. Eppig , “Intercommunication Between Mammalian Oocytes and Companion Somatic Cells,” BioEssays 13 (1991): 569–574. 1772412 10.1002/bies.950131105

[34] A. J. Levi , M. F. Raynault , P. A. Bergh , M. R. Drews , B. T. Miller , and R. T. Scott , “Reproductive Outcome in Patients With Diminished Ovarian Reserve,” Fertility and Sterility 76 (2001): 666–669. 11591396 10.1016/s0015-0282(01)02017-9

[35] S. Wang , Y. Zheng , J. Li , et al., “Single‐Cell Transcriptomic Atlas of Primate Ovarian Aging,” Cell 180 (2020): 585–600.e19. 32004457 10.1016/j.cell.2020.01.009

[36] X. Li , X. Li , and L. Deng , “Chrysin Reduces Inflammation and Oxidative Stress and Improves Ovarian Function in D‐Gal‐Induced Premature Ovarian Failure,” Bioengineered 13 (2022): 8291–8301. 35311454 10.1080/21655979.2021.2005991 PMC9161991

[37] S. Liu , Y. Jia , S. Meng , Y. Luo , Q. Yang , and Z. Pan , “Mechanisms of and Potential Medications for Oxidative Stress in Ovarian Granulosa Cells: A Review,” International Journal of Molecular Sciences 24 (2023): 9205. 37298157 10.3390/ijms24119205 PMC10252376

[38] K. Lefkimmiatis , F. Grisan , L. F. Iannucci , N. C. Surdo , T. Pozzan , and G. Di Benedetto , “Mitochondrial Communication in the Context of Aging,” Aging Clinical and Experimental Research 33 (2021): 1367–1370. 31925726 10.1007/s40520-019-01451-9

[39] Z. Zhang , L. Zhang , L. Zhou , Y. Lei , Y. Zhang , and C. Huang , “Redox Signaling and Unfolded Protein Response Coordinate Cell Fate Decisions Under ER Stress,” Redox Biology 25 (2019): 101047. 30470534 10.1016/j.redox.2018.11.005 PMC6859529

[40] O. Pluquet , A. Pourtier , and C. Abbadie , “The Unfolded Protein Response and Cellular Senescence. A Review in the Theme: Cellular Mechanisms of Endoplasmic Reticulum Stress Signaling in Health and Disease,” American Journal of Physiology‐Cell Physiology 308 (2015): C415–C425. 25540175 10.1152/ajpcell.00334.2014

[41] X. Liang , Z. Yan , W. Ma , et al., “Peroxiredoxin 4 Protects Against Ovarian Ageing by Ameliorating D‐Galactose‐Induced Oxidative Damage in Mice,” Cell Death & Disease 11 (2020): 1053. 33311472 10.1038/s41419-020-03253-8 PMC7732846

[42] S. Zhang , H. F. Zhou , Y. N. Liu , et al., “[Modified Dihuang Decoction Improves Ovarian Reserve in Mice by Regulating Bcl‐2‐Related Mitochondrial Apoptosis Pathway],” Zhongguo Zhong yao za zhi = Zhongguo zhongyao zazhi = China Journal of Chinese Materia Medica 46 (2021): 6493–6501. 34994142 10.19540/j.cnki.cjcmm.20210823.401

[43] P. Liu , E. X. Shang , D. W. Qian , Y. Zhu , Y. P. Tang , and J. A. Duan , “[Screening for Differentially‐Expressed Proteins in Ovary of Primary Dysmenorrheal Mice With Xiangfu Siwu Decoction Administration Using Nano LC‐LTQ‐Orbitrap‐MS/MS],” Zhongguo Zhong yao za zhi = Zhongguo zhongyao zazhi = China Journal of Chinese Materia Medica 41 (2016): 3060–3064. 28920349 10.4268/cjcmm20161620

[44] A. Azimi , S. M. Ghaffari , G. H. Riazi , S. S. Arab , M. M. Tavakol , and S. Pooyan , “α‐Cyperone of *Cyperus rotundus* Is an Effective Candidate for Reduction of Inflammation by Destabilization of Microtubule Fibers in Brain,” Journal of Ethnopharmacology 194 (2016): 219–227. 27353867 10.1016/j.jep.2016.06.058

[45] B. Huang , G. Hu , X. Zong , et al., “α‐Cyperone Protects Dopaminergic Neurons and Inhibits Neuroinflammation in LPS‐Induced Parkinson's Disease Rat Model via Activating Nrf2/HO‐1 and Suppressing NF‐κB Signaling Pathway,” International Immunopharmacology 115 (2023): 109698. 36634417 10.1016/j.intimp.2023.109698

[46] B. Xia , Y. Tong , C. Xia , C. Chen , and X. Shan , “α‐Cyperone Confers Antidepressant‐Like Effects in Mice via Neuroplasticity Enhancement by SIRT3/ROS Mediated NLRP3 Inflammasome Deactivation,” Frontiers in Pharmacology 11 (2020): 577062. 33132912 10.3389/fphar.2020.577062 PMC7579414

[47] K. Papadimitriou , P. Kountourakis , A. E. Kottorou , et al., “Follicle‐Stimulating Hormone Receptor (FSHR): A Promising Tool in Oncology?,” Molecular Diagnosis & Therapy 20 (2016): 523–530. 27392476 10.1007/s40291-016-0218-z

[48] D. A. Modi , S. Sunoqrot , J. Bugno , D. D. Lantvit , S. Hong , and J. E. Burdette , “Targeting of Follicle Stimulating Hormone Peptide‐Conjugated Dendrimers to Ovarian Cancer Cells,” Nanoscale 6 (2014): 2812–2820. 24468839 10.1039/c3nr05042d

[49] B. Huang , J. Liu , S. Fu , et al., “α‐Cyperone Attenuates H2O2‐Induced Oxidative Stress and Apoptosis in SH‐SY5Y Cells via Activation of Nrf2,” Frontiers in Pharmacology 11 (2020): 281. 32322198 10.3389/fphar.2020.00281 PMC7156596

[50] R. B. Daude , R. Bhadane , and J. S. Shah , “Alpha‐Cyperone Mitigates Renal Ischemic Injury via Modulation of HDAC‐2 Expression IDiabetes: Insights From Molecular Dynamics Simulations and Experimental Evaluation,” European Journal of Pharmacology 975 (2024): 176643. 38754539 10.1016/j.ejphar.2024.176643

