# Supplementary figures and images for: Biomimetic Nanoparticles Loaded With α‐Cyperone Alleviating LPS‐Induced Inflammation in KGN Cells by Activating Nrf2/HO‐1 and Suppressing ROS
Source: J Biochem Mol Toxicol. 2025 Sep 18;39(9):e70495. doi: 10.1002/jbt.70495 (PMC12445330; doi:10.1002/jbt.70495)

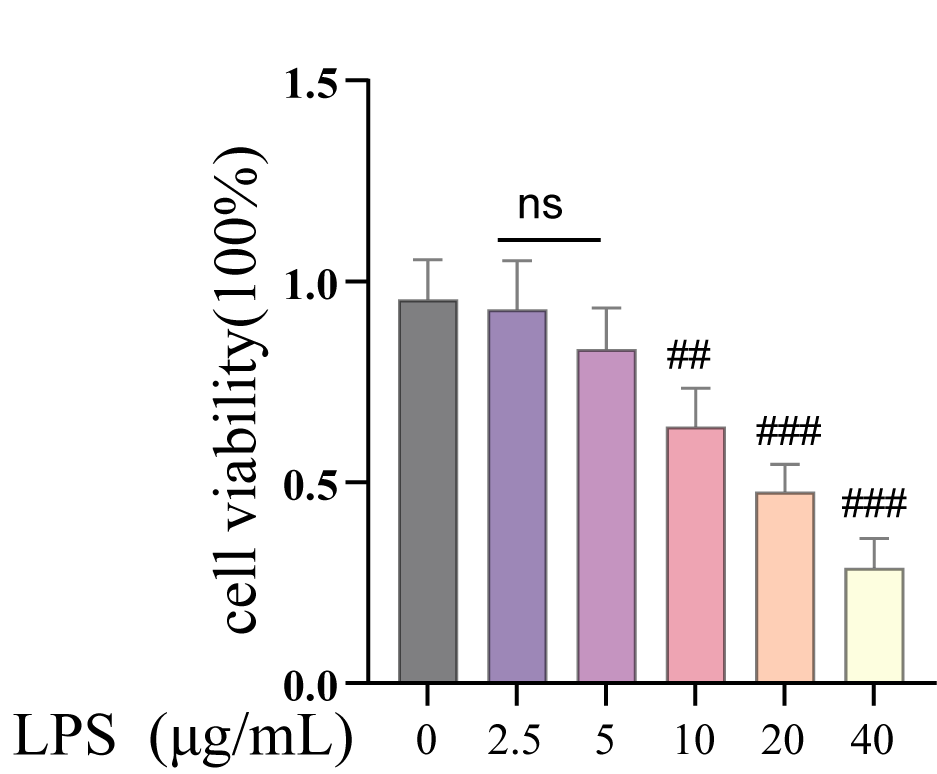

Supplement: Supplementary file 1 — Figure S1: Relative KGN cell viability levels in different LPS groups. [file JBT-39-e70495-s004.tif]

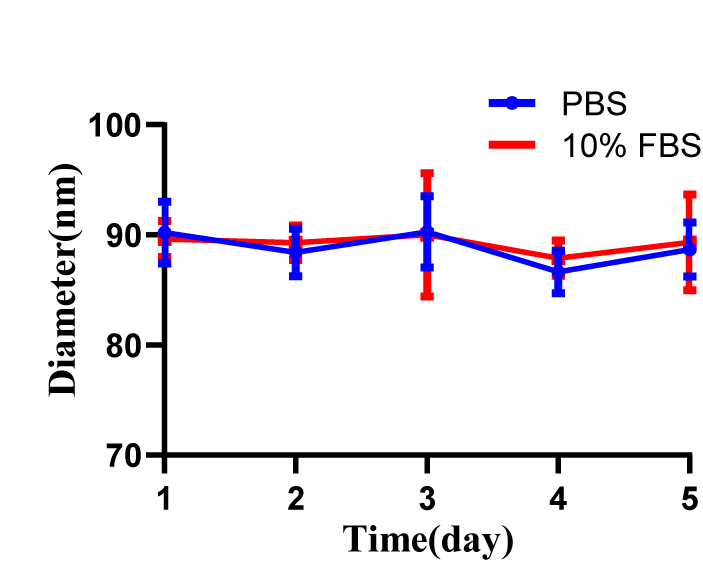

Supplement: Supplementary file 2 — Figure S2: The long‐term stability of PA NPs. [file JBT-39-e70495-s001.tif]

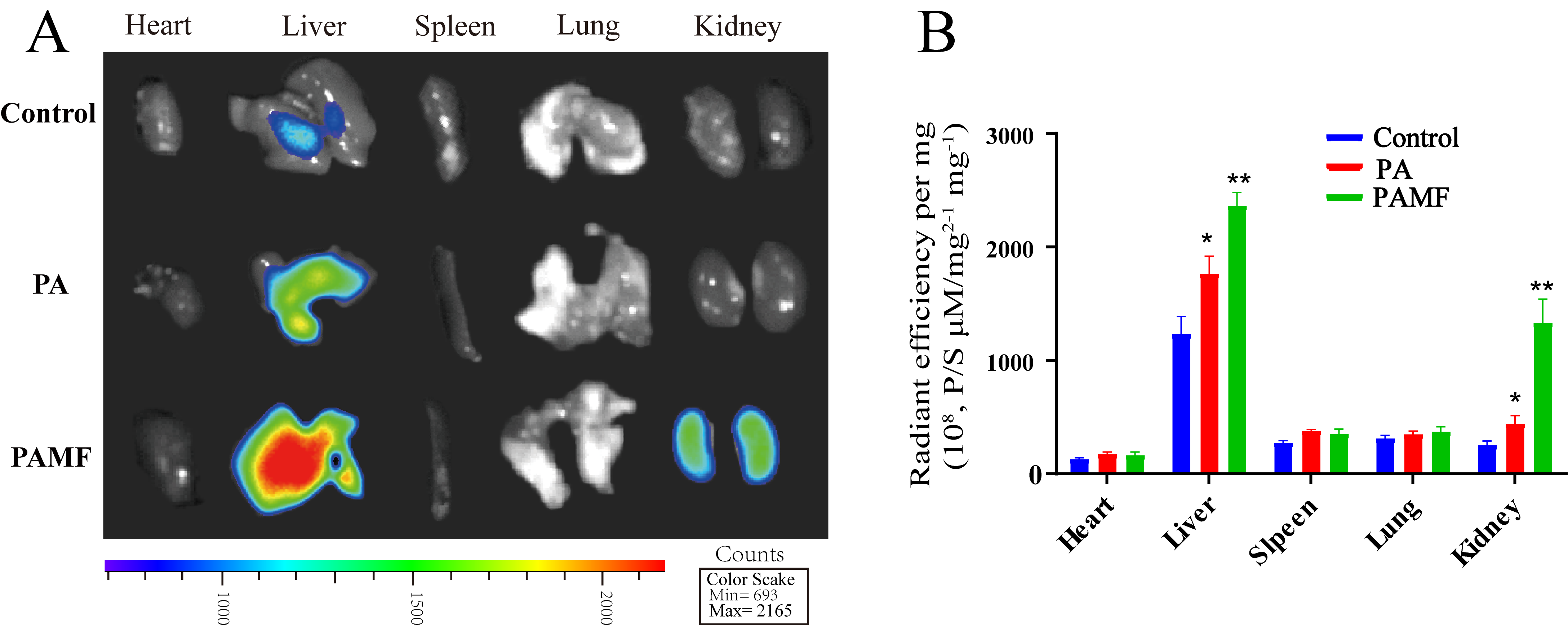

Supplement: Supplementary file 3 — Figure S3: In vivo metabolic pathways of PA NPs. [file JBT-39-e70495-s003.tif]
