# Supplementary material for: Biomimetic Nanoparticles Loaded With α‐Cyperone Alleviating LPS‐Induced Inflammation in KGN Cells by Activating Nrf2/HO‐1 and Suppressing ROS
Source: J Biochem Mol Toxicol. 2025 Sep 18;39(9):e70495. doi: 10.1002/jbt.70495 (PMC12445330; doi:10.1002/jbt.70495)
Supplement: Supplementary file 4 — supmat. [file JBT-39-e70495-s002.docx]

**Fig S1 Relative KGN cell viability levels in different LPS groups.** ^##^P < 0.01, ^###^P < 0.001 vs. control group.

**Fig S2**：**The long-term stability of PA NPs**

**Fig S3**: **In vivo metabolic pathways of PA NPs**
